# *Helicobacter pylori* and gastric cancer: time for mega-trials?

**DOI:** 10.1038/sj.bjc.6690444

**Published:** 1999-06

**Authors:** J Danesh

**Affiliations:** Clinical Trial Service Unit and Epidemiological Studies Unit, Nuffield Department of Clinical Medicine, Radcliffe Infirmary,University of Oxford, Oxford, OX2 6HE, UK


*British Journal of Cancer* (1999) **80**(7), 927–929
© 1999 Cancer Research Campaign
Article no. bjoc.1998.0444

Editorial

### *Helicobacter pylori* and gastric cancer: time for mega-trials?

**J Danesh**

Clinical Trial Service Unit and Epidemiological Studies Unit, Nuffield Department of Clinical Medicine, Radcliffe Infirmary, University of Oxford, Oxford OX2 6HE, UK

The first report of an association between chronic infection with *Helicobacter pylori*, a spiral bacterium of the stomach, and gastric cancer appeared in 1991 and in 1994 the International Agency for Research of Cancer declared *H. pylori* a human 'carcinogen' (IARC Working Group, 1994). Five years after that report, however, the causal role of *H. pylori* in gastric cancer remains controversial, with risk estimates ranging from ninefold (Webb and Forman, 1996) to no important association at all (Crespi and Citarda, 1996). Recent quantitative reviews, by contrast, suggest that *H. pylori* infection is likely to be only a moderate risk factor for gastric cancer (Danesh, 1999*a*, 1999*b*).

Reliable epidemiological evidence on *H. pylori* and gastric cancer is still relatively sparse. Although the number of cases reported in prospective studies has increased by threefold since 1994, there are now a total of only about 800 cases in ten published prospective studies. A synthesis of these studies indicates that *H. pylori* is two or three times more common in people with gastric cancer than in others (Figure 1). Doubts persist, however, about the extent to which inadequate adjustment for possible confounding factors, such as smoking and markers of poverty, and the preferential publication of studies with more extreme results might have led to exaggerated estimates. The biological plausibility of a causal association is suggested by strong correlations reported between *H. pylori* infection and putatively precancerous gastric lesions (such as atrophic gastritis and intestinal metaplasia) (Kuipers et al, 1995; Sakaki et al, 1995) and by the production of lesions that resemble human gastric cancer in Mongolian gerbils following long-term experimental infection (Watanabe et al, 1998). Even if a two- or threefold relative risk were established, however, it would not explain the sharp variations in gastric cancer mortality between populations (e.g. 20-fold higher in certain parts of China than in the USA) (Peto, 1990) or between past and present (e.g. the fivefold decrease in Scotland between 1950 and 1990) (Swerdlow et al, 1998). Moreover, although *H. pylori* infects men and women about equally and is strongly associated with duodenal ulceration, gastric cancer is twice as common in men and may be inversely associated with duodenal ulceration (Howson et al, 1986; Hansson et al, 1996). Clearly, if *H. pylori* is a cause of gastric cancer, there must be some other major cause(s) of the disease.

To assess the role of the infection in gastric cancer more precisely would require larger studies than hitherto, especially in socially homogenous populations in which residual confounders are at a minimum. Larger studies of early-onset cases (Kikuchi et al, 1995), or of *H. pylori* subtypes (e.g. cytotoxin-positive strains) (Blaser et al, 1995), or of cases with certain types of tumours (e.g. distal gastric cancers) (Parsonnet et al, 1991), may yield stronger relative risks, but previously reported studies in such subgroups have been based on small numbers and liable to biases. Moreover, some previous studies may have underestimated the role of *H. pylori* infection in gastric cancer, particularly those in developing countries that tested for antigens from Western populations (since the prevalence of *H. pylori* antigens can vary substantially between regions), and those that failed to exclude cancers reported in the first few years of follow-up (since disease itself may render some cases seronegative). Larger and better epidemiological studies may require collaborative efforts to achieve appropriate sample sizes and might benefit from the testing of hypotheses about other infective agents in gastric cancer as well (e.g. the Epstein–Barr virus, the genome of which is present in some gastric cancers) (Hsieh et al, 1998). More detailed combined analyses of the existing data on *H. pylori*, perhaps based on individual participant data from each of the prospective studies, could help to allow more complete adjustment for other risk factors and to assess associations in particular subgroups.

Unlike several other persistent infective agents that cause > 20-fold relative risks for particular cancers (Danesh et al, 1997*b*), the available evidence suggests that *H. pylori* is a comparatively moderate risk factor for gastric cancer (Table 1). But, if even a causal twofold increased risk could be confirmed, it would suggest an effect that would be large enough to be of some practical relevance, as *H. pylori* would then be responsible for about one-quarter of all gastric cancers, or about 250 000 such deaths worldwide each year (Murray and Lopez, 1997). This relatively large attributable risk derives from the high prevalence of *H. pylori*, which is found in the stomachs of about one-third of the adults in developed countries (where it has been decreasing in prevalence) and about two-thirds of the adults in developing countries (Goodwin et al, 1997).

The current trials of anti-bacterial interventions in gastric cancer prevention may well be inadequate to assess reliably any such moderate effects. Five randomized trials of *H. pylori* eradication in the prevention of gastric cancer are in progress worldwide, and they aim collectively to randomize about 25 000 *H. pylori* seropositive individuals by the year 2005, with a weighted mean age at entry of about 50 and weighted mean follow-up of about 10 years (Forman, 1998). Two-thirds are to be recruited in Western European countries where the incidence of gastric cancer is very low until old age, and one-third in China and Japan.

*Received 17 November 1998*
*Accepted 1 December 1998*

*Correspondence to:* J Danesh

**Table 1** Examples of common persistent infective agents associated with cancers

| Year identified | Infective agent | Associated cancer | Approximate relative risk (infected vs uninfected) |
|---|---|---|---|
| 1965 | Hepatitis B virus | Liver cancer | > 50 |
| 1983 | *Helicobacter pylori* | Gastric adenocarcinoma | 2.5 |
| 1983 | Human papillomavirus (mainly types 16 and 18) | Cervical cancer | 20 |
| 1994 | Human herpesvirus-8 | Kaposi's sarcoma | >50 |

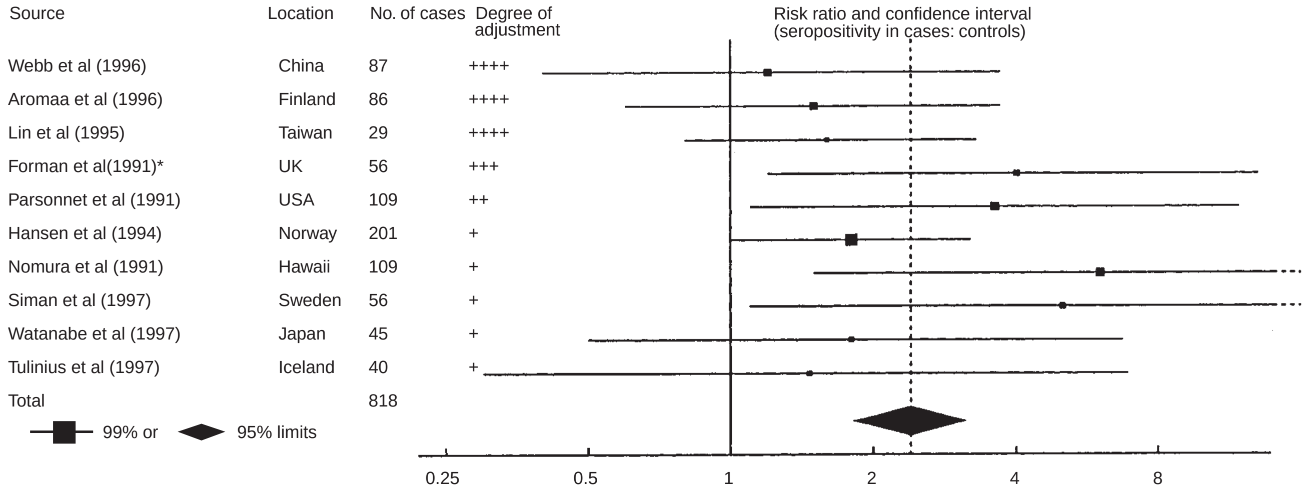

**Figure 1** Risk ratios in prospective seroepidemiological studies of *H. pylori* and gastric cancer. Black squares are proportional to the number of cases, with horizontal lines representing confidence intervals. Degree of adjustment denoted as + for age and sex only; ++ these and smoking; +++ these and markers of poverty; ++++ these and dietary information. See Danesh (1998) for details. *Updated by Wald et al (1997)

However, even if *H. pylori* eradication can reduce the eventual incidence of gastric cancer by 25%, some future combined analysis of all these trials currently in progress might well provide a false negative result. This is suggested by analogy with smoking cessation, which takes many years to produce a large proportional reduction in lung cancer. Smoking cessation at age 60 produces a reduction of less than 50% in lung cancer mortality during the first decade after stopping, but produces a reduction of more than 50% during the second decade (Halpern et al, 1993). If, by analogy, the reductions in gastric cancer during the first and second decades after allocation to *H. pylori* eradication are taken to be 25% and 50%, respectively, then a trial in the UK of *H. pylori* eradication at age 60 might have to randomize at least 100 000 individuals with at least 1 decade of follow-up to achieve statistically reliable results. Indeed, an even larger number might be needed if there is a delay of some years before benefits emerge, if antibiotic treatments become more widely used for 'non-ulcer dyspepsia' or if gastric cancer rates fall more steeply than anticipated (Danesh et al, 1996).

Recruitment of such a large total number of individuals for a gastric prevention trial in one or more studies might be feasible, but only with simplified study designs. This might involve, for example, recruitment at a single visit, at which individuals are randomized irrespective of antibody status (although a blood sample should be stored for future testing) and then followed up to ascertain cases of cancer and causes of death. Such a blanket strategy might even have logistical and scientific advantages to only recruiting the elderly in the UK, seropositive for *H. pylori* antibodies. For example, storing baseline blood samples for future testing could allow use of any improved assay methods available only at the end of the trial (including novel techniques to identify different bacterial subtypes). Moreover, given that individuals in late middle-age are much more likely to develop gastric cancer within 10–20 years of randomization than younger people, an efficient research strategy might involve 'factorial' designs, where bacterial eradication regimens are introduced into existing trials of unrelated interventions among individuals of appropriate age already monitored for long-term follow-up (e.g. participants in vascular disease prevention trials). As effective vaccines against *H. pylori* are not available (Michetti et al, 1996), perhaps some trials should also collect information on the overall risks (Blaser, 1997), costs (Parsonnet et al, 1996; Sonnenberg and Inadomi, 1998) and possible benefits (Danesh et al, 1997*a*) of a short course of antibiotic treatment.

The launch of some such mega-trials may well be justified, for it is difficult to see how important clinical questions (e.g. 'Does killing *H. pylori* in middle-aged adults reduce the eventual incidence of gastric cancer?') and health policy questions (e.g. 'What are the overall risks and benefits for the population of widespread *H. pylori* eradication?') can be reliably answered without them.

### ACKNOWLEDGEMENTS

Richard Doll commented helpfully on this manuscript. I acknowledge the support of a Merton College Junior Research Fellowship and the Frohlich Trust.

 © 1999 Cancer Research Campaign

### REFERENCES

Blaser MJ (1997) Not all *Helicobacter pylori* strains are created equal: should all be eliminated? *Lancet* **349**: 1020–1022

Blaser MJ, Perez-Perez GI and Kleanthous H (1995) Infection with *Helicobacter pylori* strains possessing cagA is associated with an increased risk of developing adenocarcinoma of the stomach. *Cancer Res* **55**: 2111–2115

Crespi M and Citarda F (1996) *Helicobacter pylori* and gastric cancer: an overrated risk? *Scand J Gastroenterol* **31**: 1041–1046

Danesh J (1999) Is *Helicobacter pylori* a cause of gastric neoplasia? In *Infections and Cancer,* Beral V, Newton R and Weiss R (eds). Cold Springs Harbor Press: New York. In Press

Danesh J (1999) *Helicobacter pylori* and gastric cancer: systematic review of the epidemiological evidence. *Aliment Pharm Ther*. In Press

Danesh J, Forman D, Collins R and Peto R (1996) *Helicobacter pylori* screening and gastric cancer. *Lancet* **348**: 758–759

Danesh J, Collins R and Peto R (1997*a*) Chronic infections and coronary heart disease: is there a link? *Lancet* **350**: 430–436

Danesh J, Newton R and Beral V (1997*b*) A human germ project? *Nature* **389**: 21–24

Forman D (1988) Lessons from ongoing intervention studies. In Helicobacter pylori: *Basic Mechanisms to Clinical Cure*, Hunt R and Tytgat G (eds) Kluwer: Amsterdam

Goodwin CS, Mendall MM and Northfield TC (1997) *Helicobacter pylori* infection. *Lancet* **349**: 265–269

Halpern MT, Gillespie BW and Warner KE (1993) Patterns of absolute risk of lung cancer mortality in former smokers. *J Natl Cancer Inst* **85**: 457–464.

Hansson LE, Nyren O, Hsing AW, Bergstrom R, Josefsson S, Chow WH, Fraumeni JF and Adami HO (1996) The risk of stomach cancer in patients with gastric or duodenal ulcer disease. *N Engl J Med* **335**: 242–249

Howson CP, Hiyama T and Wynder EL (1986) The decline in gastric cancer: epidemiology of an unplanned triumph. *Epidemiol Rev* **8**: 1–27

Hsieh LL, Lin PJ, Chen TC and Ou JT (1998) Frequency of Epstein–Barr virus-associated gastric adenocarcinoma in Taiwan. *Cancer Lett* **129**: 125–129

IARC Working Group (1994) Monographs on the evaluation of the carcinogenic risks to humans: schistosomes, liver flukes and *Helicobacter pylori*. IARC **61**: 177–241

Kikuchi S, Wada O, Nakajima T, Nishi T, Kobayashi O, Konishi T and Inaba Y (1995) Serum anti-*Helicobacter pylori* antibody and gastric carcinoma among young adults. *Cancer* **75**: 2789–2793

Kuipers EJ, Uyterlinde AM, Pena AS, Roosendaal R, Pais G, Nelis GF, Festen HPM and Meuwissen SGM (1995) Long-term sequelae of *Helicobacter pylori* gastritis. *Lancet* **345**: 1525–1528

Michetti P, Wadstrom T, Kraehenbuhl JP, Lee A, Kreiss C and Blum AL (1996) Frontiers in *Helicobacter pylori* research: pathogenesis, host response, vaccine development and new therapeutic approaches. *Eur J Gastroenterol Hepatol* **8**: 717–722

Murray CJL and Lopez AD (1997) Alternative projections of mortality and disability by cause 1990–2020: Global Burden of Disease Study. *Lancet* **349**: 1498–1504

Parsonnet J, Vandersteen D, Goates J, Sibley RK, Pritikin J and Chang Y (1991) *Helicobacter pylori* infection in intestinal- and diffuse-type adenocarcinomas. *J Natl Cancer Inst* **83**: 640–643

Parsonnet J, Harris RA, Hack HM and Owens DK (1996) Modelling cost-effectiveness of *Helicobacter pylori* screening to prevent gastric cancer: a mandate for clinical trials. *Lancet* **348**: 150–154

Peto R (1990) General reflections on principles and purposes: indirect and direct purposes of the present monograph. In Junshi C, Campbell TC, Junyao L and Peto R (eds), pp. 72–78. *Diet, Lifestyle and Mortality in China: A Study of the Characteristics of 65 Chinese Counties*. Oxford University Press: Oxford

Sakaki N, Momma K, Egawa N, Yamada Y, Kan T and Ishiwata J (1995) The Influence of *Helicobacter pylori* infection on the progression of gastric mucosal atrophy and occurrence of gastric cancer. *Eur J Gastroenterol Hepatol* **7**: S59–S62

Sonnenberg A and Inadomi JM (1998) Review article: Medical decision models of *Helicobacter pylori* therapy to prevent gastric cancer. *Aliment Pharm Ther* **12**: 111–121

Swerdlow AJ, Dos Santos Silva I, Reid A, Qiao Z, Brewster DH and Arrundale J (1998) Trends in cancer incidence and mortality in Scotland. *Br J Cancer* **77**: 1–16

Watanabe T, Tada M, Nagai H, Sasaki S and Nakao M (1998) *Helicobacter pylori* infection induces gastric cancer in Mongolian gerbils. *Gastroenterology* **115**: 642–648

Webb PM and Forman D (1996) *Helicobacter pylori* as a risk factor for cancer. *Baillière’s Clin Gastroenterol* **9**: 563–582

*© 1999 Cancer Research Campaign*

